# Defects of Hearing, and Other Evils

**Published:** 1877-10-20

**Authors:** 


Defects of Hearing and other evils; the Result of Enlarged or Hypertrophied Tonsils, and the urgent necessity of immediate and proper treatment, &c.—By Prof. A. W. Calhoun, M. D., Atlanta, Ga. A valuable paper from transactions of Medical Association of Georgia.

